# Supplementary figures and images for: Cysteine Rich Intestinal Protein 2 is a copper-responsive regulator of skeletal muscle differentiation and metal homeostasis
Source: PLoS Genet. 2024 Dec 5;20(12):e1011495. doi: 10.1371/journal.pgen.1011495 (PMC11671023; doi:10.1371/journal.pgen.1011495)

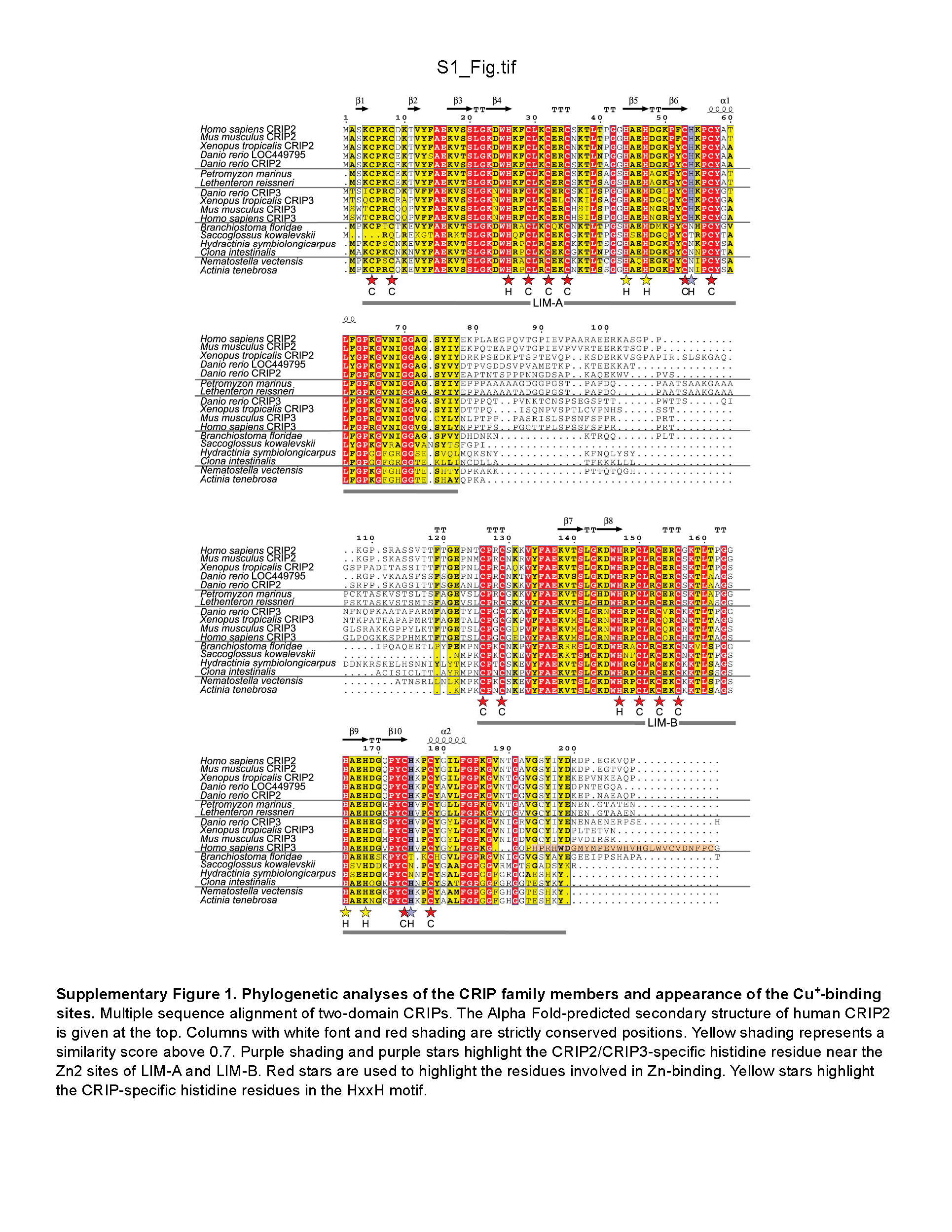

Supplement: S1 Fig — (TIF) [file pgen.1011495.s001.tif]

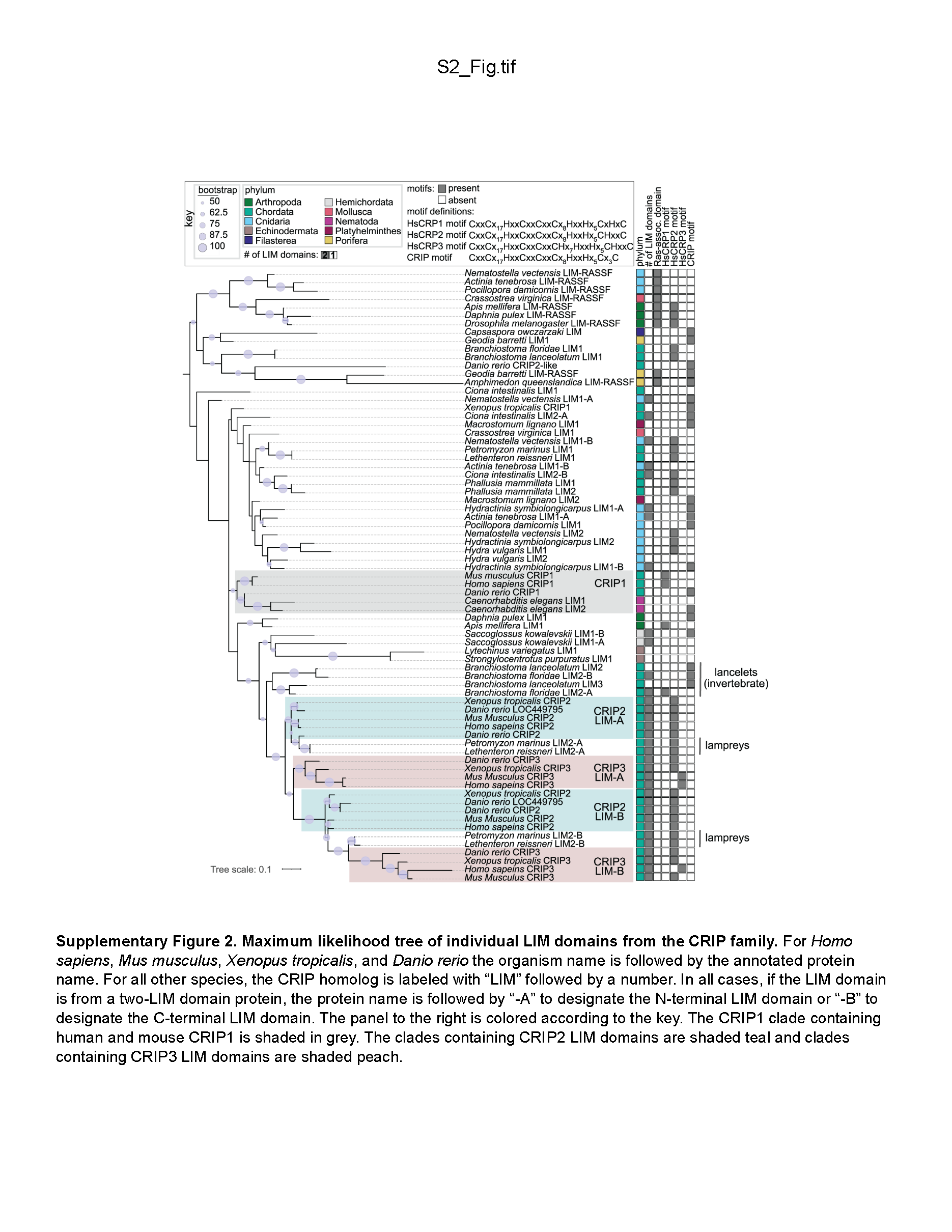

Supplement: S2 Fig — (TIF) [file pgen.1011495.s002.tif]

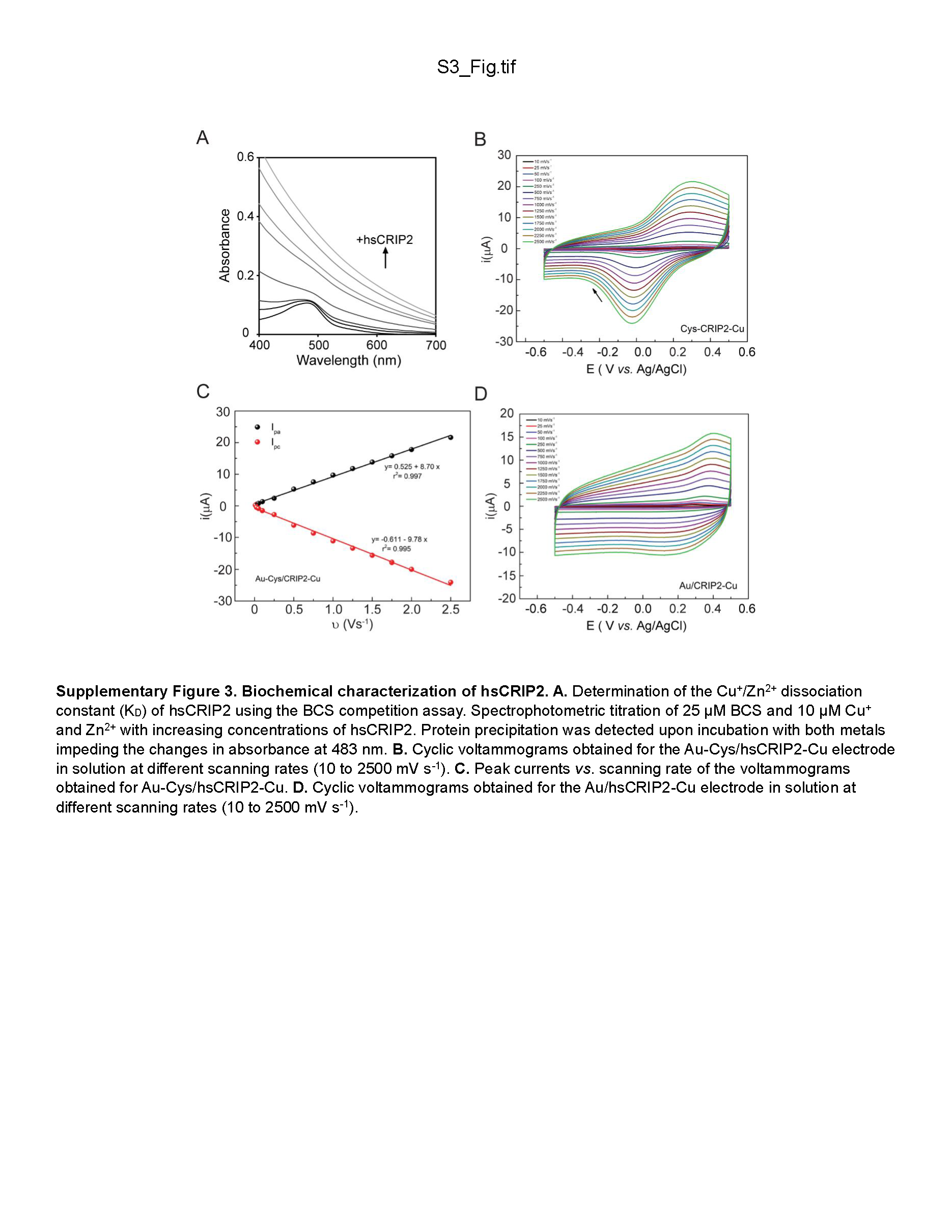

Supplement: S3 Fig — (TIF) [file pgen.1011495.s003.tif]

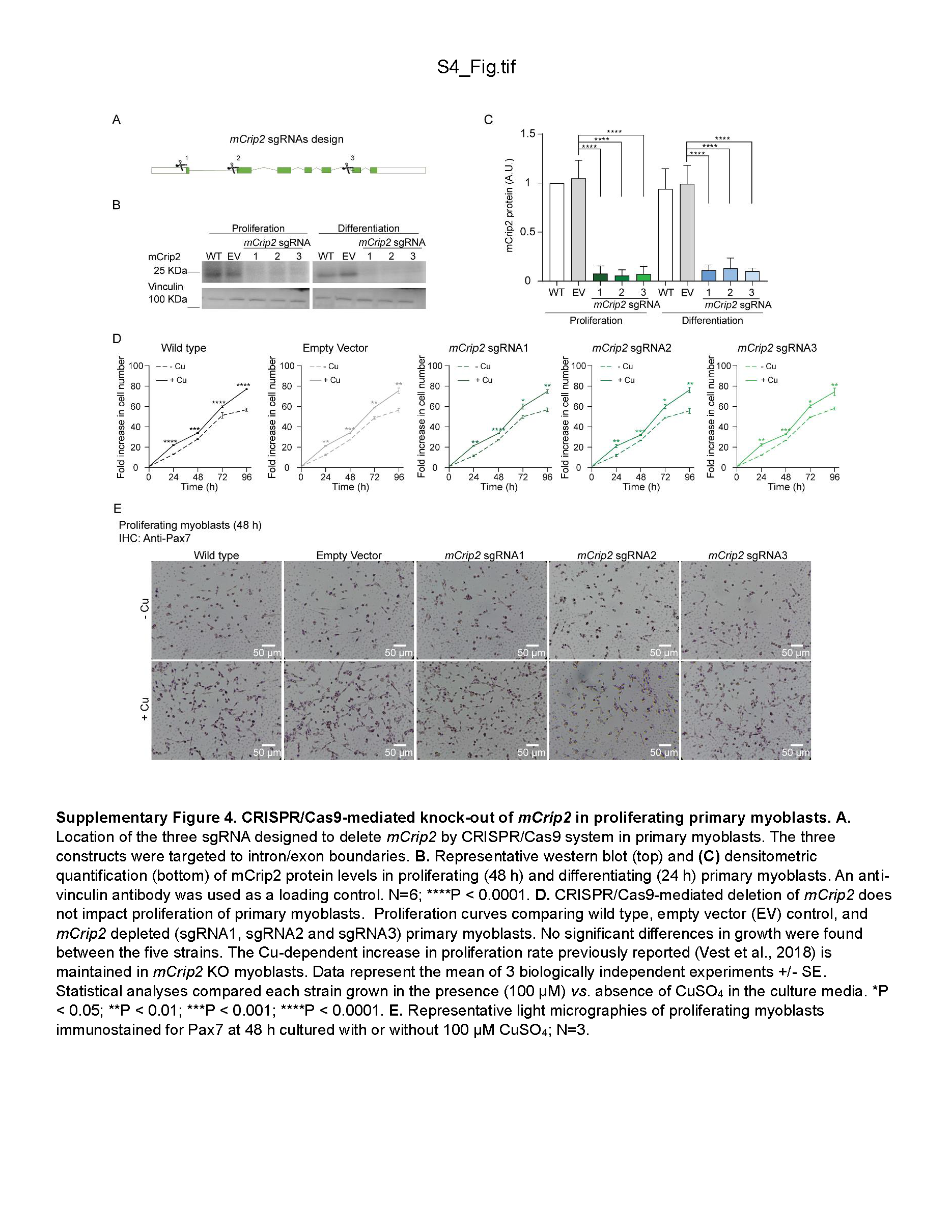

Supplement: S4 Fig — (TIF) [file pgen.1011495.s004.tif]

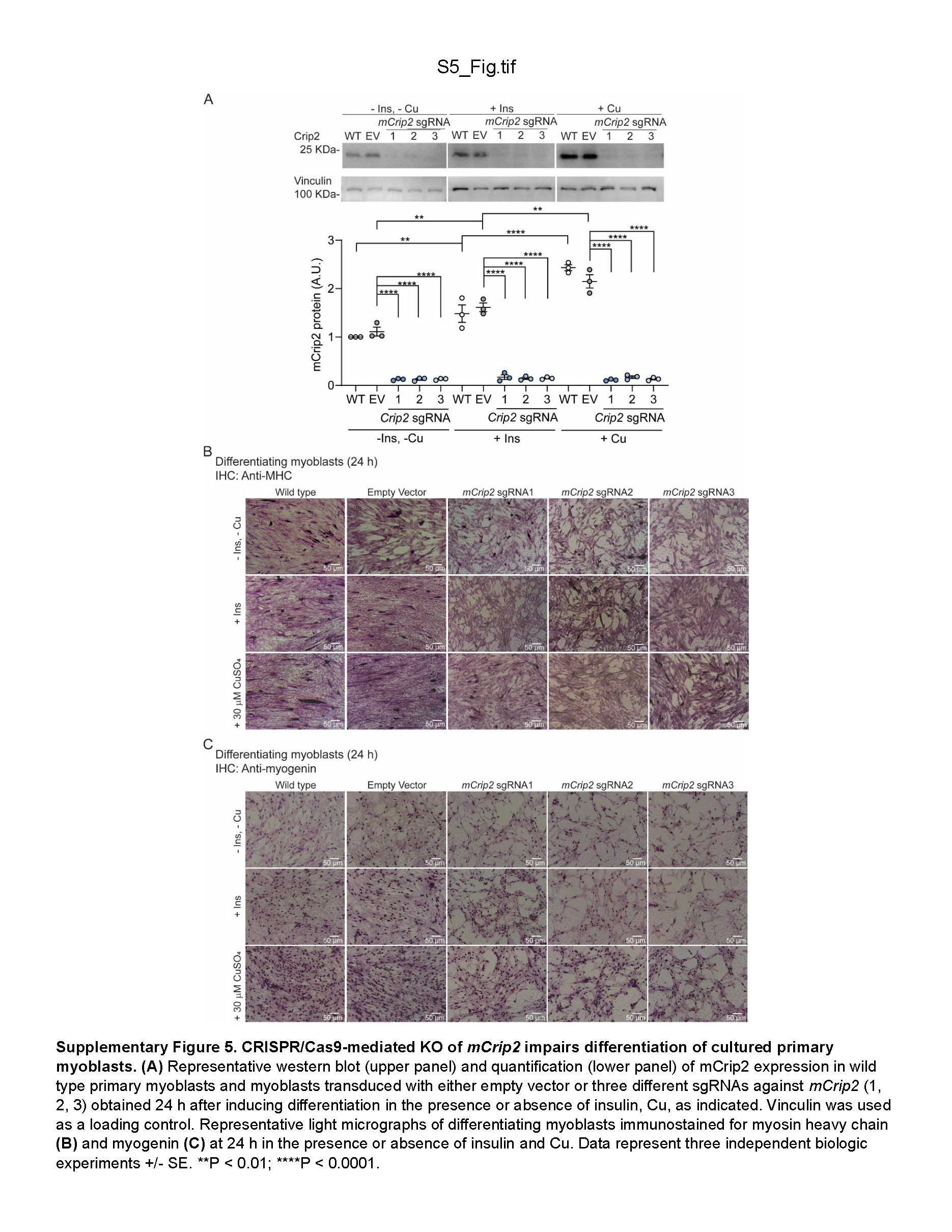

Supplement: S5 Fig — (TIF) [file pgen.1011495.s005.tif]

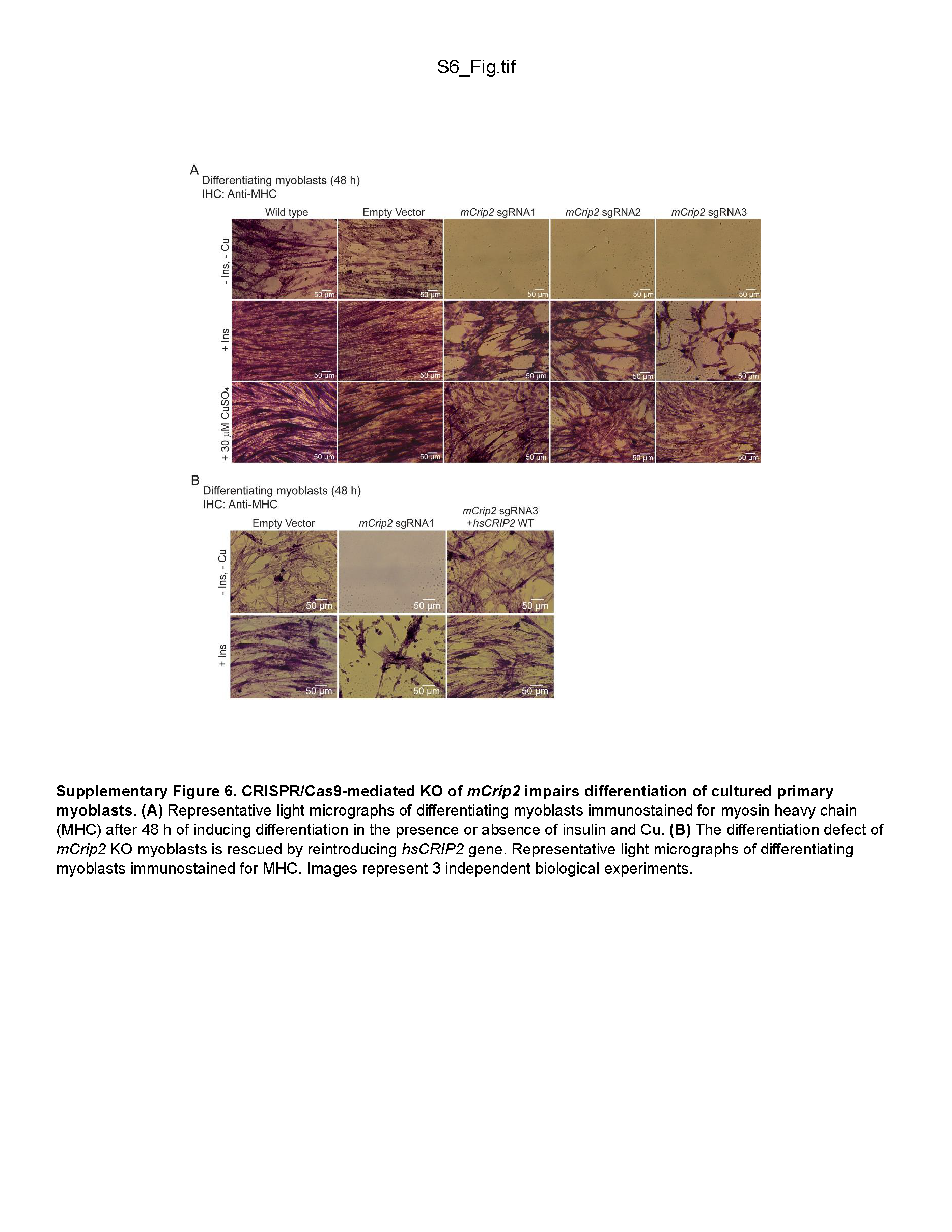

Supplement: S6 Fig — (TIF) [file pgen.1011495.s006.tif]

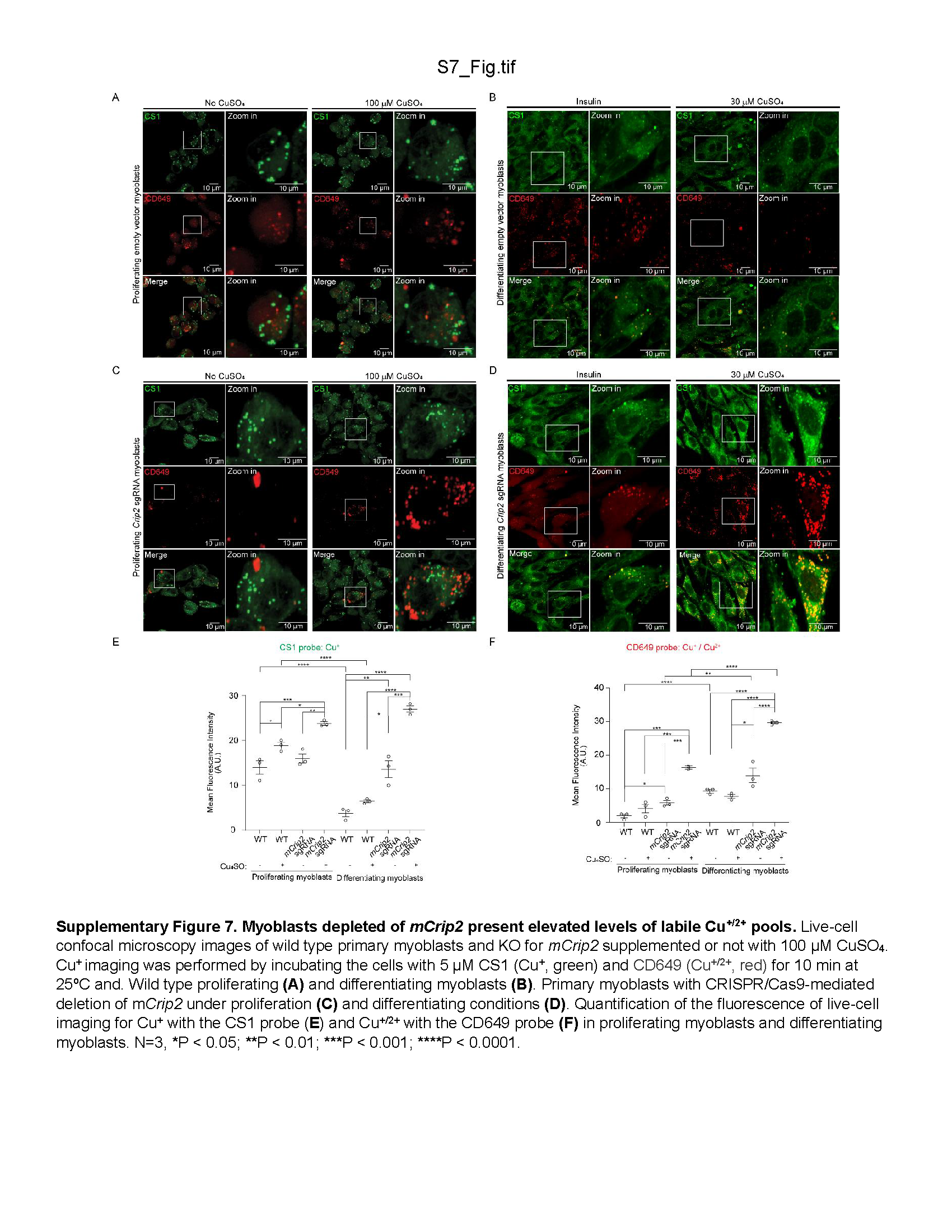

Supplement: S7 Fig — (TIF) [file pgen.1011495.s007.tif]

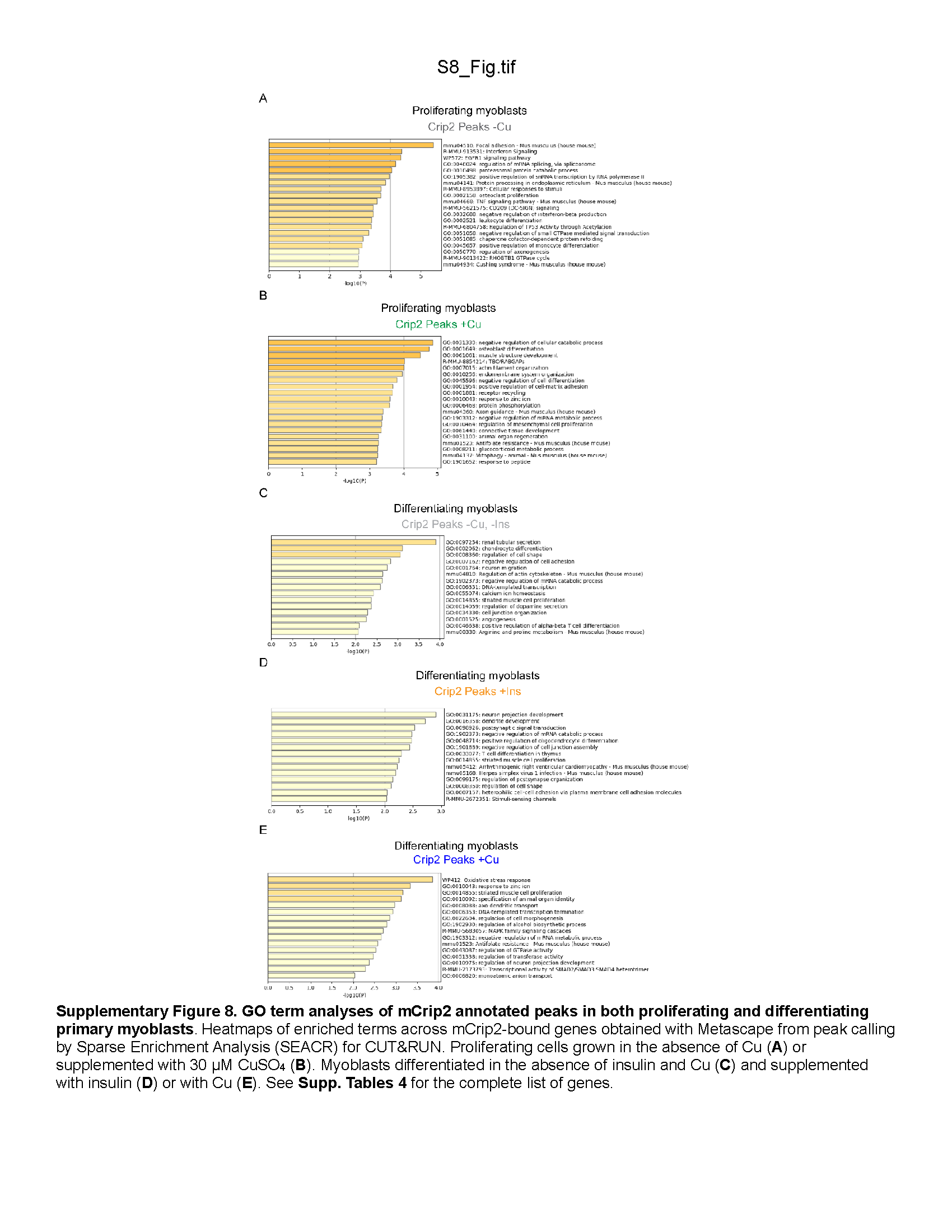

Supplement: S8 Fig — (TIF) [file pgen.1011495.s008.tif]

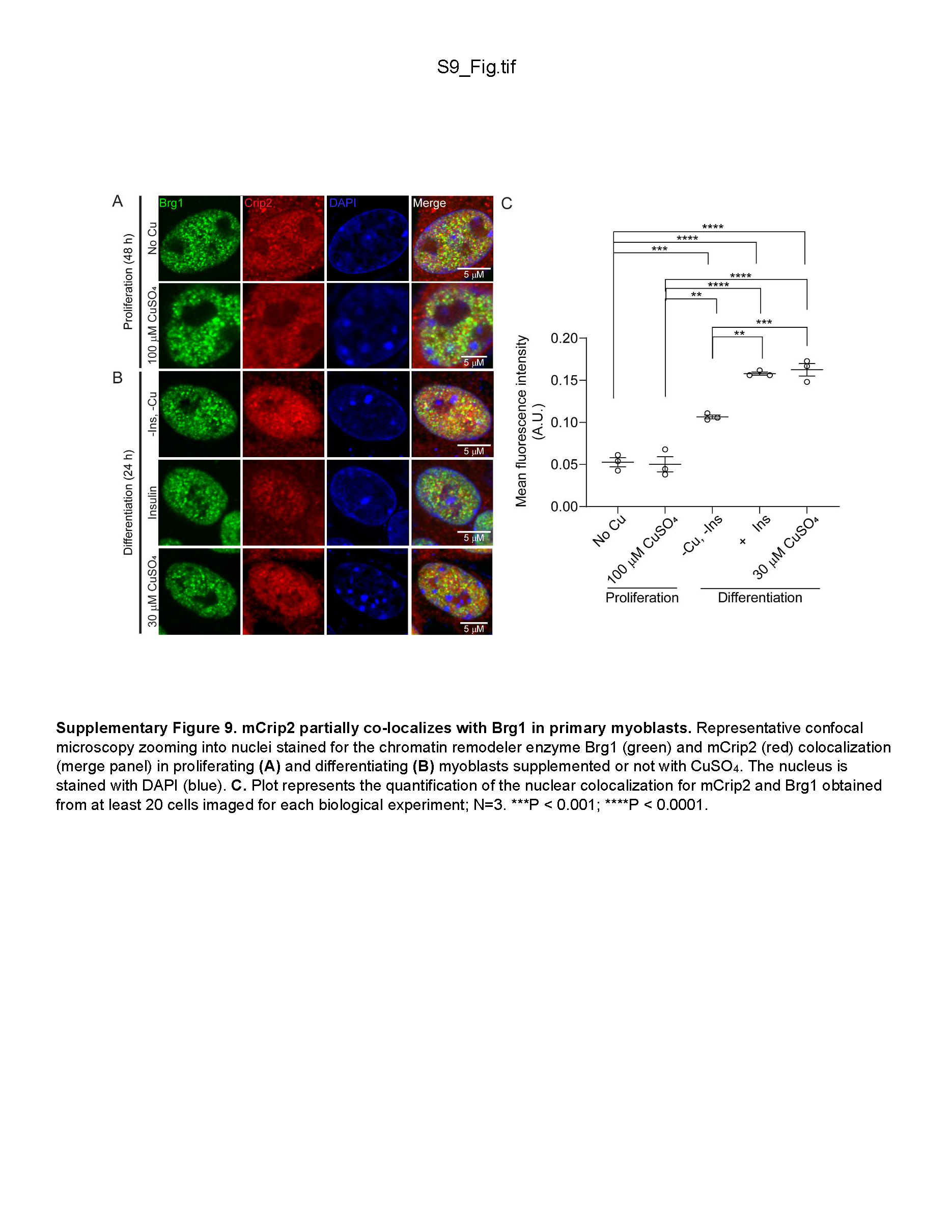

Supplement: S9 Fig — (TIF) [file pgen.1011495.s009.tif]

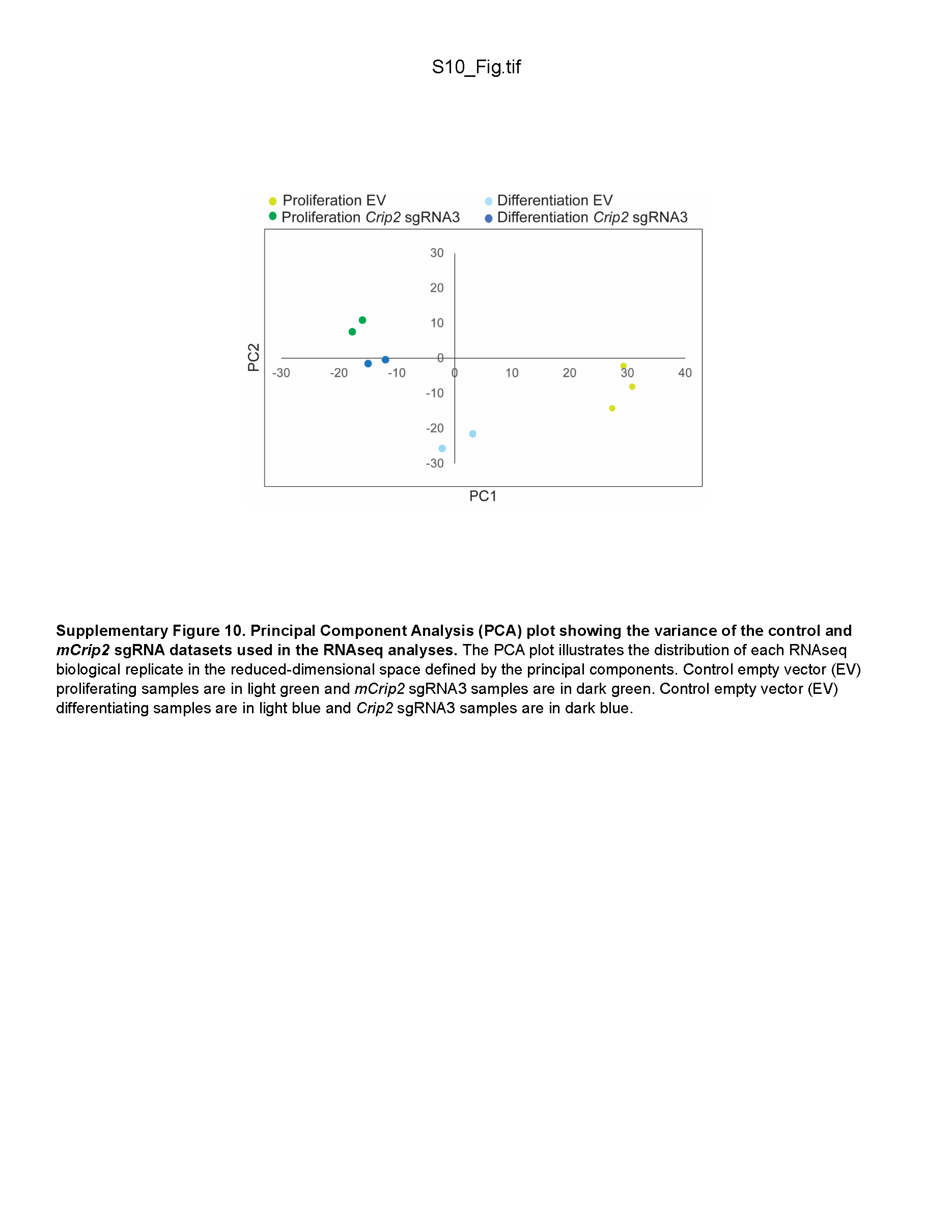

Supplement: S10 Fig — (TIF) [file pgen.1011495.s010.tif]

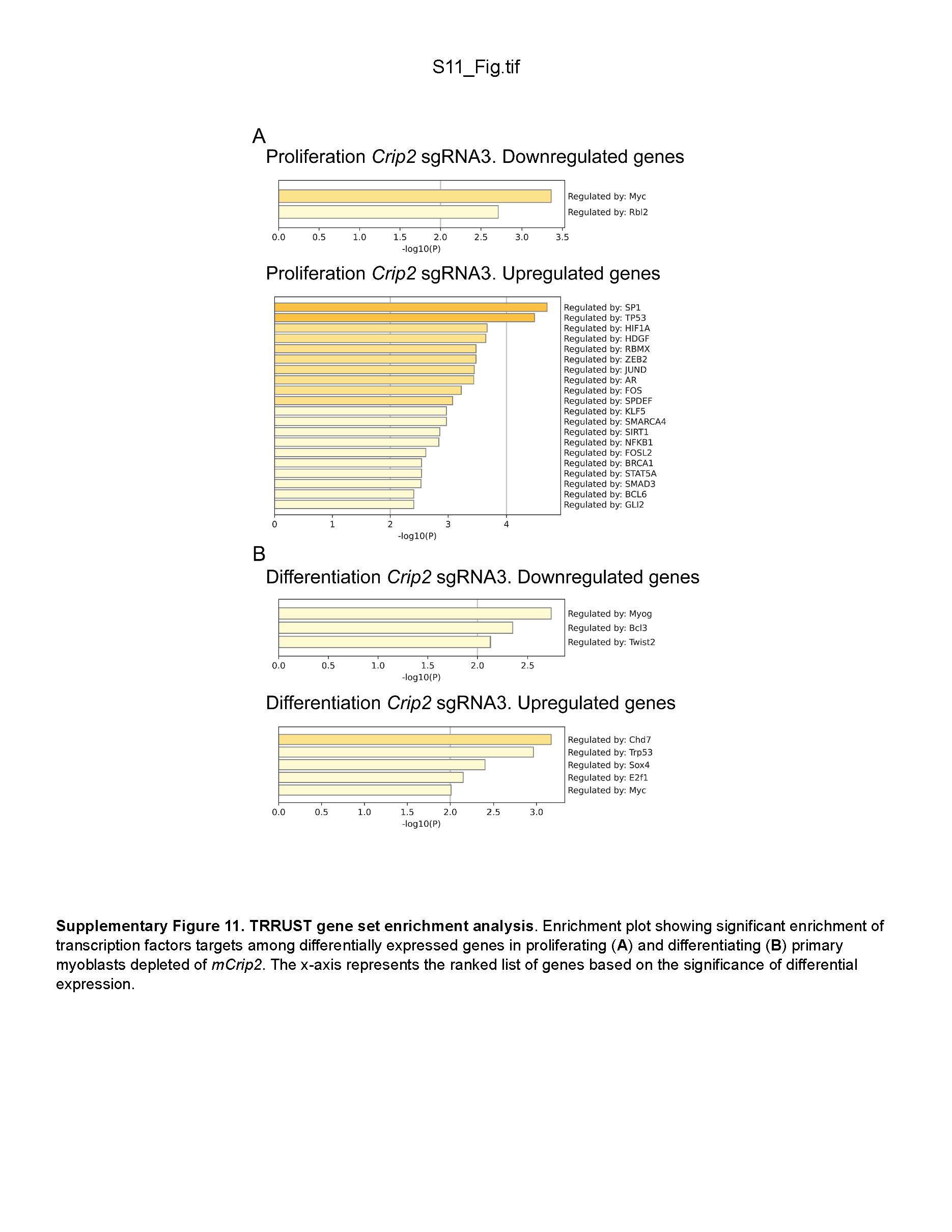

Supplement: S11 Fig — (TIF) [file pgen.1011495.s011.tif]

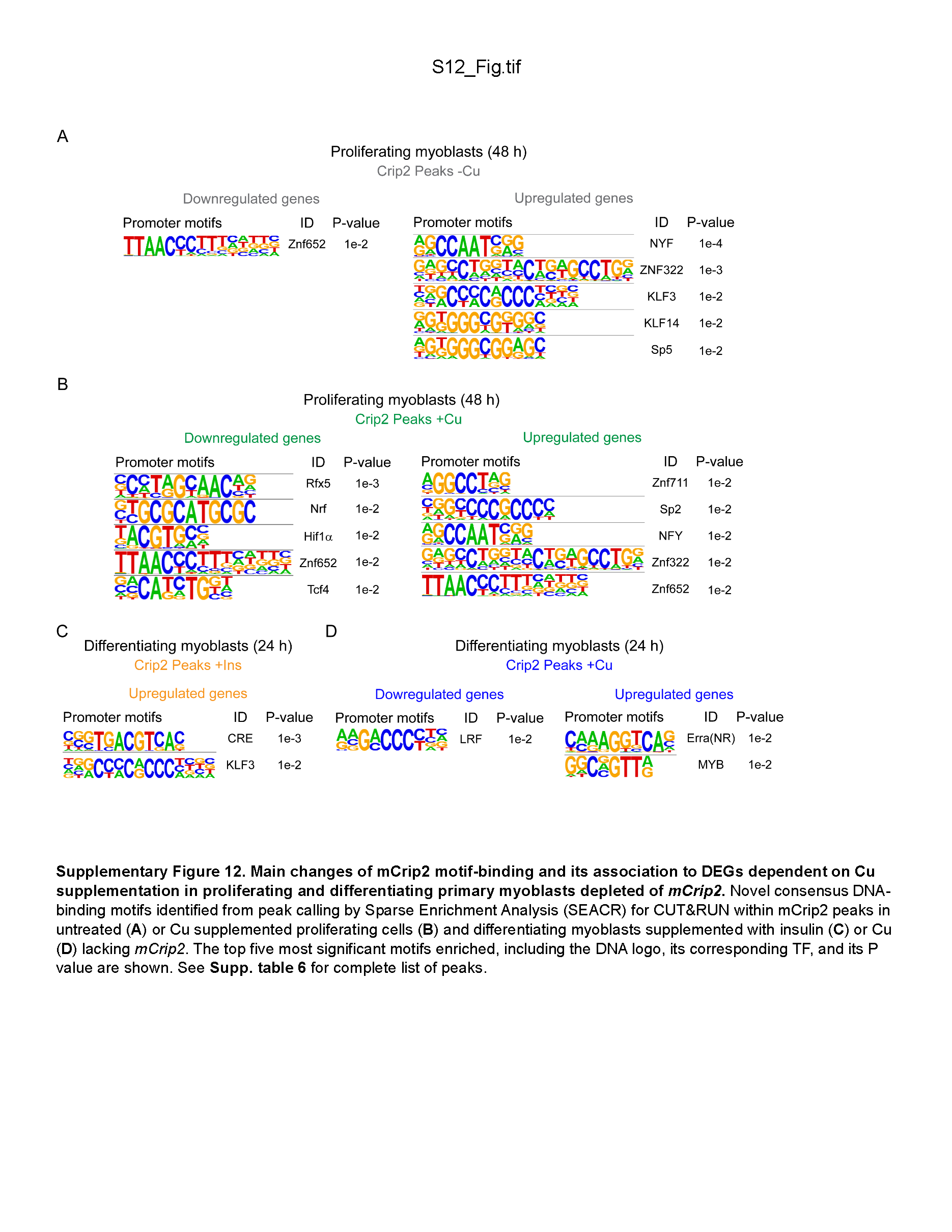

Supplement: S12 Fig — (TIF) [file pgen.1011495.s012.tif]
