# Supplementary material for: Cysteine Rich Intestinal Protein 2 is a copper-responsive regulator of skeletal muscle differentiation and metal homeostasis
Source: PLoS Genet. 2024 Dec 5;20(12):e1011495. doi: 10.1371/journal.pgen.1011495 (PMC11671023; doi:10.1371/journal.pgen.1011495)
Supplement: S2 Table — (PDF) [file pgen.1011495.s014.pdf]

S2_Table

List of primers used in this study.

Primer Name	5' Sequence	3' sequence	Use
Crip2	GAATTCGCCTCCAAGTGTCCC	CCATGGTTGGGCTGAACTGTGCCTTC	cloning into pR-RIBA and pBABE
Crip2 sgRNA1	CACCGGGACACTTGGAGGCCATGGT	AAACACCATGGCCTCCAAGTGTCCC	sgRNAs for CRISPR/Cas9
Crip2 sgRNA2	CACCGCTTCCTCTCTACAGCTGAGA	AAACTCTCAGCTGTAGAGAGGAAGC	sgRNAs for CRISPR/Cas9
Crip2 sgRNA3	CACCGTAGGGCTGGCCATCGTGCTG	AAACCAGCACGATGGCCAGCCCTAC	gRNAs for CRISPR/Cas9
q-Eef1A	GGCTTCACTGCTCAGGT GATTATC	ACACATGGGCTTGCCAGGGAC	Gene Expression
q-Pax7	GCAGCTGGAGGAGCTAGAGAAG	GTCTCCTGGCTTGATGGAGTC	Gene expression
q-Myog	CAAGTGTGCACATCTGTTCTAGTCTCT	GTATCATCAGCACAGGAGACCTTGGT	Gene expression
q-Mck	GCCGGGGATGAGGAGTCCTAC	GCAGTGCGGAGGCAGAGTGTA	Gene expression
q-Mtf1	AGATGATATTGTCGTCTGGACTGTG	GAAGCGGAAGTGACGCTAGGGACAG	Gene expression
q-RpsA	GGTGGCACCAACCTTGACTTTC	GTCAGCAGGATTCTCGATGGCA	Gene expression
q-Rps8	GGAGGCAATAAGAAGTACCGTGC	TTGGTGCGGACAAGCTCGTTGT	Gene expression
ch-MyoD1	GCTCAGCAACTATGCTCTACA	CGCCCTCCAAAGCGCACAAAT	ChIP-qPCR
ch-Mt1	CTCCGCCCGAAAAGTGCGCTC	GAAGCTGGAGCTACGGAGTAA	ChIP-qPCR
ch-Mt2	TTAGCACACAAGACATGC	TTTCTCCCGAGTCCCTT	ChIP-qPCR
