# Supplementary material for: Cysteine Rich Intestinal Protein 2 is a copper-responsive regulator of skeletal muscle differentiation and metal homeostasis
Source: PLoS Genet. 2024 Dec 5;20(12):e1011495. doi: 10.1371/journal.pgen.1011495 (PMC11671023; doi:10.1371/journal.pgen.1011495)
Supplement: S3 Table — (PDF) [file pgen.1011495.s015.pdf]

S3_Table

**Parameters obtained from Cyclic voltammetry at different scan rates for Au bare and cysteine modified electrodes with hsCRIP2 protein.**

| Protein | E° (mV vs Ag/AgCl) | $\Delta E_p$ (mV) | $k_s$ ($s^{-1}$) | α | Γ ($mol^* cm^{-2}$) |
|---|---|---|---|---|---|
| [a]Au-Cys/CRIP2-Cu | 151 | 295 | 1.02 | 0.53 | $1.07x10^{-10}$ |
| [a]Au/CRIP2-Cu | n.c | n.c | n.c | n.c | n.c. |
| [b]Au/Cu-SOD | n.c | n.c | n.c | n.c | n.c. |
| [b]Au-Cys/Cu-SOD1 | 88 | 213 | 1.05 | 0.54 | $4.28x10^{-11}$ |

n.c. *not calculated*, due the absence of either the oxidation or reduction signal.
[a] hsCRIP2 cyclic voltammetry parameters.
[b] For comparison: Published values obtained for SOD1 by Jimenez-Gonzalez *et al.*, 2023

**Reference**

M.L. Jimenez-Gonzalez JJG-G, R. Antano-Lopez , L. Ortiz-Frade. Thermodynamic study of superoxide dismutase adsorption processes over cysteine-gold electrode. Electrochimica Acta. 2023;440(141677). doi: 10.1016/j.electacta.2022.141677.
